# Ebola virus protein VP40 stimulates IL-12– and IL-18–dependent activation of human natural killer cells

**DOI:** 10.1172/jci.insight.158902

**Published:** 2022-08-22

**Authors:** Hung Le, Paul Spearman, Stephen N. Waggoner, Karnail Singh

**Affiliations:** 1 Division of Infectious Diseases, Department of Pediatrics, Cincinnati Children’s Hospital Medical Center, Cincinnati, Ohio, USA.; 2 Department of Pediatrics, University of Cincinnati College of Medicine, Cincinnati, Ohio, USA.; 3 Division of Infectious Diseases, Department of Pediatrics, Emory University School of Medicine, Atlanta, Georgia, USA.; 4 Division of Human Genetics, Department of Pediatrics, Cincinnati Children’s Hospital Medical Center, Cincinnati, Ohio, USA.

**Keywords:** Immunology, Infectious disease, Innate immunity, NK cells

## Abstract

Accumulation of activated natural killer (NK) cells in tissues during Ebola virus infection contributes to Ebola virus disease (EVD) pathogenesis. Yet, immunization with Ebola virus-like particles (VLPs) comprising glycoprotein and matrix protein VP40 provides rapid, NK cell–mediated protection against Ebola challenge. We used Ebola VLPs as the viral surrogates to elucidate the molecular mechanism by which Ebola virus triggers heightened NK cell activity. Incubation of human peripheral blood mononuclear cells with Ebola VLPs or VP40 protein led to increased expression of IFN-γ, TNF-α, granzyme B, and perforin by CD3^–^CD56^+^ NK cells, along with increases in degranulation and cytotoxic activity of these cells. Optimal activation required accessory cells like CD14^+^ myeloid and CD14^–^ cells and triggered increased secretion of numerous inflammatory cytokines. VP40-induced IFN-γ and TNF-α secretion by NK cells was dependent on IL-12 and IL-18 and suppressed by IL-10. In contrast, their increased degranulation was dependent on IL-12 with little influence of IL-18 or IL-10. These results demonstrate that Ebola VP40 stimulates NK cell functions in an IL-12– and IL-18–dependent manner that involves CD14^+^ and CD14^–^ accessory cells. These potentially novel findings may help in designing improved intervention strategies required to control viral transmission during Ebola outbreaks.

## Graphical abstract

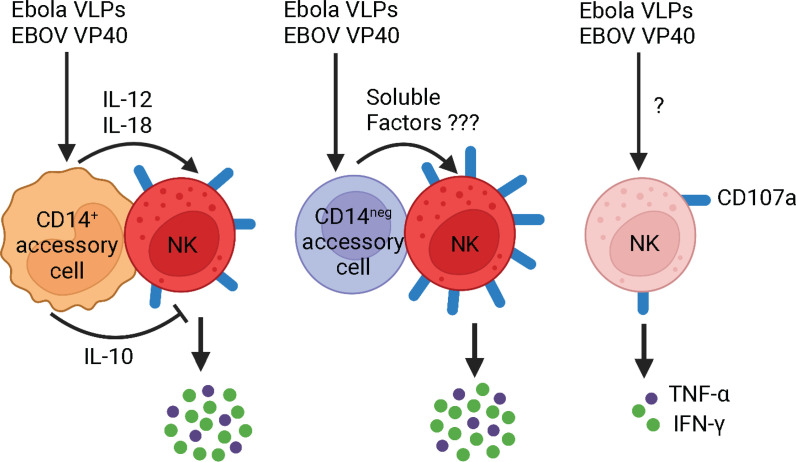

## Introduction

Natural killer (NK) cells are important components of the innate immune system, tasked with the role of detecting and eliminating pathogen-infected and aberrantly transformed cells (1). Their activity is tightly regulated through the integration of signals downstream of an array of inhibitory and activating NK cell receptors. Upon activation, they release preformed perforin- and granzyme-containing cytotoxic granules into the contact area between the NK and the target cells. The secreted perforin undergoes oligomerization to form pores in the target cell membrane through which granzymes can pass and induce apoptosis in target cells (2–5). In addition to cytotoxic functions, NK cells also modulate functions of other immune cells through the secretion of cytokines including IFN-γ and TNF-α (6). Many viruses have developed strategies to avoid activating NK cells, thus evading their detection and subsequent elimination by these cells (7–9).

Ebola viruses, the causative agents of Ebola virus disease (EVD), are filamentous, negative-sense, single-stranded RNA viruses belonging to family Filoviridae. Since Ebola viruses’ discovery in 1976, 6 species with varying degrees of lethality have been identified. They include Zaire Ebola virus (EBOV), Sudan virus (SUDV), Bundibugyo virus (BDBV), Tai-Forest virus (TAFV), Reston Ebola virus, and Bombali Ebola virus, named after the geographical locations they were first discovered in. Structurally, Ebola viruses consist of an inner nucleocapsid made up of nucleoprotein and containing the viral RNA, transcription factor VP30, cofactor VP35, and RNA polymerase L. This nucleocapsid is surrounded by a lipid bilayer that is covered abundantly with Ebola glycoprotein (GP) spikes. Viral proteins, VP40 and VP24, constitute the particle matrix and are located between the viral envelope and nucleocapsid (10). Full-length Ebola GP is a trimer of heterodimers (GP1 and GP2) present on the surface of the virions and is critical for the initial viral attachment, fusion, and entry into the target cells (11, 12). Because of its integral role in EVD pathogenesis, this protein has been the target of most vaccines and therapeutics being developed against Ebola viruses (13–15). VP40 is the major matrix protein of EBOV and coordinates virion assembly and budding. VP40 dimers at the infected cell plasma membrane reorganize into hexamers that are critical for the viral assembly and egress (16–18). Additionally, VP40 can assemble into octameric conformations that play an important role in virion replication (19, 20).

There have been more than 30 outbreaks of EVD to date, with the largest being the 2013 to 2016 Ebola outbreak in West Africa, which resulted in over 28,600 infections and 11,325 deaths (21, 22). The size and impact of this outbreak led national and international public health agencies to fast-track the development of several experimental Ebola vaccines and therapeutic countermeasures. Since then, several of these countermeasures have been evaluated in clinical trials for their safety and efficacy (14, 23–38). A replication-competent vesicular stomatitis virus–based Ebola vaccine (VSV-ZEBOV) has been approved by the US Food and Drug Administration (23, 27, 32) while vaccines based on nonreplicating platforms, like adenoviruses, have shown good safety and immunogenicity profiles (24–26, 28–31, 33–35). However, no protective efficacy data in the setting of an Ebola outbreak have been reported so far for any of the vaccine candidates on nonreplicating platforms. Despite the tremendous progress made in recent years, there is still no Ebola vaccine that provides durable protective immunity against the 4 Ebola viruses that are pathogenic in humans, namely EBOV, SUDV, BDBV, and TAFV. In addition to the vaccines, several anti-EBOV GP monoclonal antibodies and Ebola RNA-dependent RNA polymerase inhibitor–based therapeutics have also been evaluated in clinical trials, with Inmazeb (a cocktail of 3 monoclonal antibodies) and Ebanga (a single monoclonal antibody) receiving Food and Drug Administration approval to treat EVD caused by EBOV (36–42).

The rapid onset and severity of EVD suggest that infections with Ebola viruses can quickly overwhelm the host innate immune system. A better understanding of the role played by innate immune cells during the early stages of Ebola virus infection will be very helpful in developing fast-acting therapeutics that could modulate innate responses and help in eliminating the virus at the first line of defense itself. Ebola virus–induced functional paralysis of the innate immune cells and their subsequent inability to activate B and T lymphocytes is considered central to the immune suppression observed in fatal cases of EVD (43, 44). Interferon-inhibiting domains present on Ebola proteins VP24 and VP35 may be involved in mediating this functional impairment (45, 46). Past studies revealed a decline in the number and function of NK cells in fatal cases of EVD (46–51). In animal models of EVD, a decrease in peripheral NK cell number was observed concomitant with their accumulation in Ebola virus–infected tissues, where they were shown to contribute to viral pathogenesis through their cytotoxic actions (52). In addition to contributing to tissue damage, the accumulated NK cells actively participated in the elimination of tissue-infiltrating lymphocytes, further aggravating the disease (52). However, the precise mechanism of NK cell activation during Ebola virus infection is still not clear.

Immunization of mice and nonhuman primates with Ebola virus-like particles (VLPs) has been reported to protect against subsequent challenge with lethal doses of Ebola viruses (53–56). This Ebola VLP-mediated protection required both innate and adaptive arms of the immune system (53, 54, 56, 57). The rapid innate protection induced by Ebola VLPs was dependent on the activation of NK cells and involved cytolysis of the Ebola-infected cells by the activated NK cells (56, 58). Surprisingly, studies from the rodent model of EVD suggested that the main component of the Ebola VLPs responsible for NK cell–dependent protection against Ebola virus was EBOV VP40, not the viral GP (56).

In the current study, we sought to elucidate the molecular mechanism by which Ebola virus or viral gene products trigger activation of NK cells. We show that EBOV VP40 stimulated cytokine secretion and cytotoxic functions of NK cells through IL-12 and IL-18, as well as cell-cell contact-dependent mechanisms. Accessory cells, including CD14^+^ myeloid cells, played an integral role in VP40-mediated NK cell stimulation. These observations further our understanding of the innate immune responses during Ebola virus infections and can inform the design of novel anti-Ebola countermeasures.

## Results

### Generation of Ebola VLPs.

**Figure 1:**
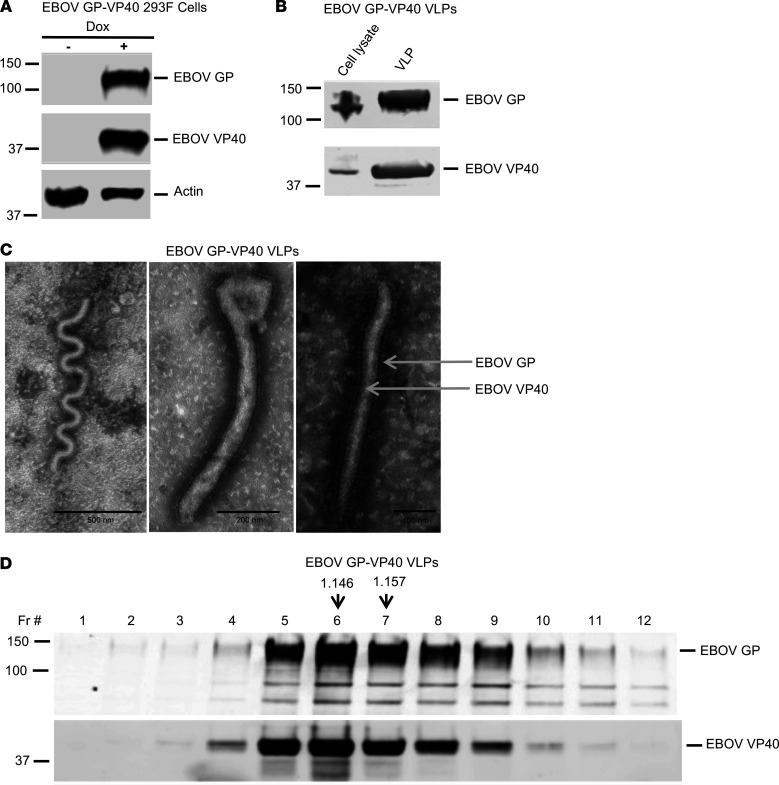
Generation of EBOV GP-VP40 293F stable cell line and characterization of EBOV GP-VP40 VLPs (Ebola VLPs). (**A**) EBOV GP-VP40 293F cells were cultured with or without doxycycline (Dox) for 24 hours and cell lysates probed for EBOV GP, EBOV VP40, or actin by Western blotting using specific antibodies. A representative Western blot is shown. (**B**) Cell lysates and VLPs collected from EBOV GP-VP40 293F cell cultures induced with doxycycline for 40 hours were probed by Western blotting using anti-EBOV GP and anti-EBOV VP40 antibodies. The majority of the EBOV GP and EBOV VP40 were secreted out of the cells as part of the VLPs. (**C**) Negative stain electron microscopy analysis of EBOV GP-VP40 VLPs showed filamentous particles of different shapes and sizes, covered abundantly with EBOV GP spikes. Scale bars are indicated with black lines (from left to right, 500 nm, 200 nm, and 100 nm). (**D**) Buoyancy density analysis of EBOV GP-VP40 VLPs. Ebola VLPs fractionated on 20% to 60% sucrose density gradient were analyzed by Western blotting for EBOV GP and EBOV VP40. Buoyance density of each fraction was calculated by measuring its refractive index. Buoyancy densities of the fractions enriched for Ebola VLPs (identified by the relative EBOV GP and VP40 contents) are shown with vertical arrows above those fractions. Numbers at left of blots indicate kilodaltons.

We manufactured Ebola VLPs by stably transfecting 293F cells with plasmids encoding tetracycline-inducible *EBOV GP* or *EBOV VP40* gene (Supplemental Figure 1; supplemental material available online with this article; https://doi.org/10.1172/jci.insight.158902DS1). These cells, labeled EBOV GP-VP40 293F cells, expressed EBOV GP and VP40 following overnight induction with doxycycline (Figure 1A), thereby facilitating spontaneous formation and secretion of VLPs into culture media. Isolated VLPs were abundantly enriched for the EBOV GP and VP40 proteins (Figure 1B). At the end of the incubation period, the majority of the newly synthesized EBOV GP and VP40 were packaged into the secreted VLPs, with very little left in the cells (Figure 1B). Negative staining electron microscopic analysis revealed that Ebola VLPs comprised heterogeneous filamentous particles, 400 to 1500 nm in size, that were abundantly covered with EBOV GP spikes (Figure 1C). Their buoyant densities ranged between 1.14 and 1.16 g/mL (Figure 1D). The Ebola protein contents in different VLP lots varied between 90 and 122 μg/mL of EBOV GP and 40 and 76 μg/mL of EBOV VP40, resulting in a GP/VP40 ratio of 1.9 ± 0.51 (Supplemental Table 1).

### Cytokine secretion by human NK cells in response to Ebola VLPs.

**Figure 2:**
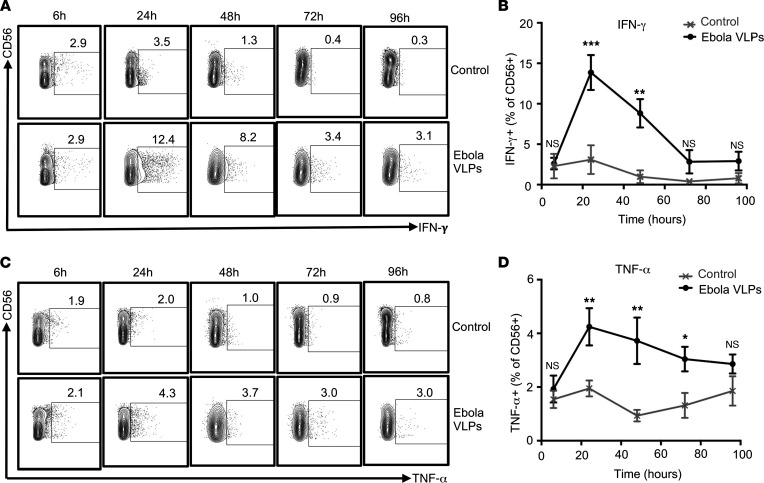
Enhanced frequencies of cytokine-secreting NK cells upon stimulation of human PBMCs with Ebola VLPs. PBMCs were stimulated with or without Ebola VLPs (final concentration: 10 μg/mL of EBOV GP) for 6, 24, 48, 72, and 96 hours. Surface markers and intracellular cytokine expression were assessed by flow cytometry. Strategy for gating single and live CD3^–^CD56^+^ NK cells is shown in Supplemental Figure 2. (**A** and **C**) Representative dot blots showing CD3^–^CD56^+^ NK cells plotted for IFN-γ and TNF-α, respectively. (**B** and **D**) Summary data from 11 independent experiments performed with PBMCs from 11 (24 and 48 hours) or 5 (6, 72, and 96 hours) independent donors are shown for IFN-γ and TNF-α, respectively. Results are shown as mean ± SEM. *P < 0.05, **P < 0.01, ***P < 0.001, as calculated by 2-tailed paired Student’s *t* test.

Freshly isolated PBMCs from different healthy donors were stimulated with Ebola VLPs, and intracellular cytokine expression by NK cells was analyzed by flow cytometry. Analysis was limited to singlet, live CD3^–^CD56^+^ NK cells (Supplemental Figure 2), which expressed IFN-γ (Figure 2, A and B) and TNF-α (Figure 2, C and D) at different time points after stimulation. The expression of cytokines by NK cells peaked 24 hours after stimulation and remained detectable up to 48 hours. The effect on IFN-γ secretion was more pronounced than on TNF-α secretion (Figure 2). Thus, Ebola VLPs provoke enhanced cytokine secretion by human NK cells.

### Cytotoxic features of human NK cells in response to Ebola VLPs.

**Figure 3:**
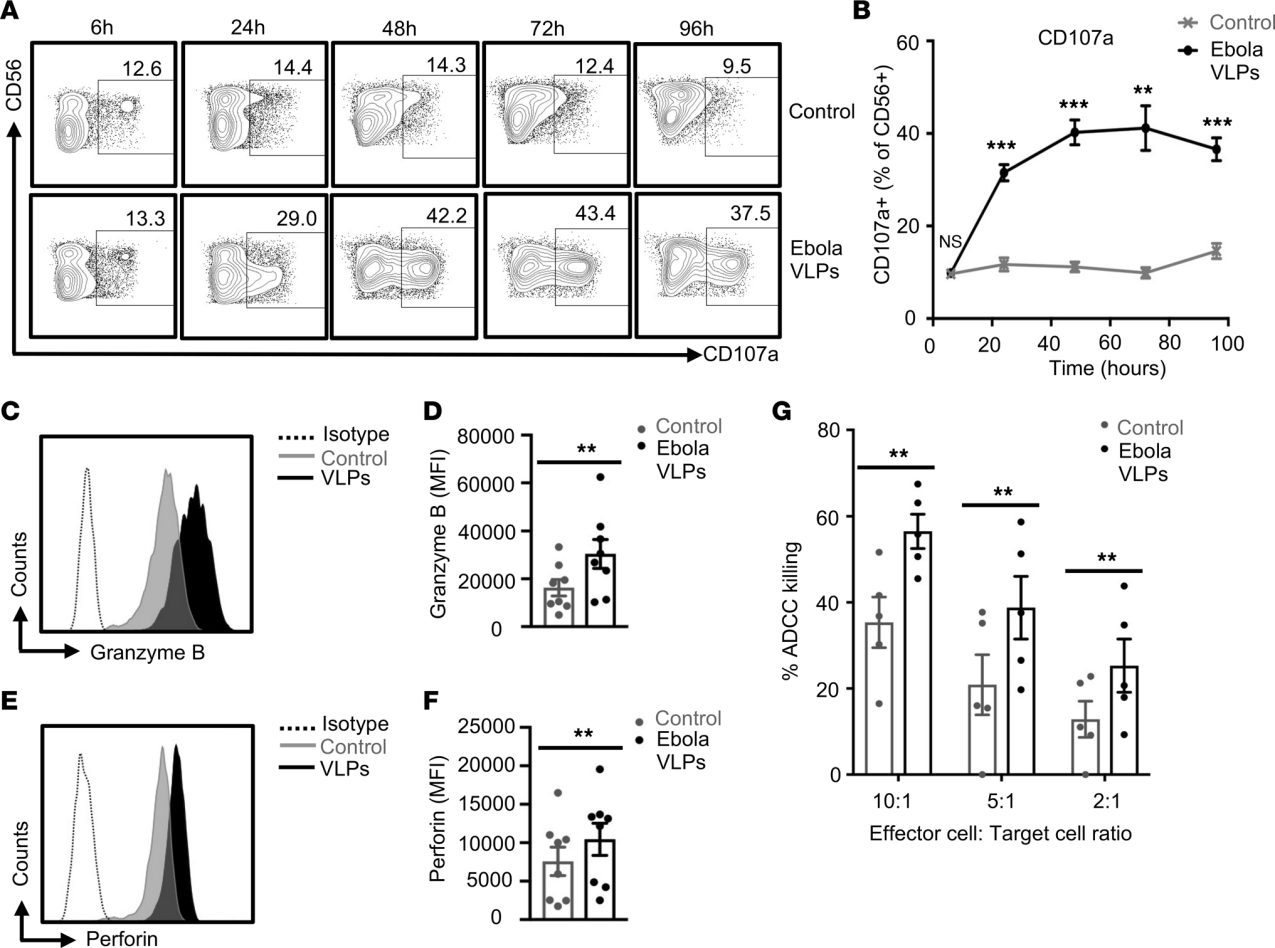
Degranulation and cytotoxic function of NK cells are enhanced by exposure to Ebola VLPs. PBMCs were stimulated with or without Ebola VLPs as in Figure 2. Cells were stained for the surface and cytotoxicity markers and data acquired and analyzed as described in Methods. Only single and live CD3^–^CD56^+^ NK cells were included in the analysis. Representative dot blots (**A**) and the corresponding summary data from 11 independent experiments performed with PBMCs from 11 (24 and 48 hours) or 5 (6, 72, and 96 hours) independent donors (**B**) showing NK cells’ degranulation (CD107a surface expression) are shown. (**C**–**F**) Representative histograms for intracellular granzyme B (**C**) and perforin (**E**) expression in gated CD3^–^CD56^+^ NK cells from PBMC cultures stimulated with Ebola VLPs (black) for 48 hours are shown. NK cells from PBMCs left unstimulated are shown in gray. Dotted histograms represent corresponding isotype controls. **D** (granzyme B) and **F** (perforin) show corresponding summary data from 8 independent experiments performed with PBMCs from 8 independent donors. (**G**) ADCC killing of target cells mediated by either unexposed (gray circles) or Ebola VLP exposed (black circles) PBMCs. PBMCs exposed to Ebola VLPs for 48 hours were analyzed for their cytotoxic potential by mixing them with the target cells expressing EBOV GP and in the presence of anti-EBOV GP plasma. Combined data from 5 independent experiments with PBMCs from 5 donors are shown. Results are shown as mean ± SEM. **P < 0.01, ***P < 0.001, as calculated by 2-tailed paired Student’s *t* test.

We assessed the cytolytic activity of NK cells in response to Ebola VLPs by flow cytometric capture of cell surface exposed CD107a because of degranulation (59). Ebola VLPs stimulated a substantial fraction of NK cells to degranulate (CD107a^+^) during the 96-hour culture period (Figure 3, A and B). Ebola VLP treatment did not alter the frequencies of granzyme B^+^perforin^+^CD3^–^CD56^+^ NK cells (Supplemental Figure 3). However, Ebola VLP treatment did significantly enhance the intracellular expression levels of both granzyme B and perforin (Figure 3, C–F). To test whether enhanced degranulation and cytolytic mediators’ expression reflect elevated cytotoxic capacity of Ebola VLP-stimulated NK cells, we employed an Ebola antibody-dependent cell-mediated cytotoxicity (ADCC) assay previously developed in our laboratory (60). EBOV GP-expressing 293 Trex target cells were incubated with rhesus macaque anti-EBOV GP plasma prior to coculture with control or Ebola VLP-exposed PBMCs. PBMCs exposed to Ebola VLPs demonstrated significantly greater capacity for ADCC killing of the target cells than the unexposed PBMCs, at all the effector/target ratios tested (Figure 3G). Thus, Ebola VLPs stimulate robust cytolytic functionality in human NK cells.

### Both CD56^bright^ and CD56^dim^ NK cells respond to Ebola VLPs.

**Figure 4:**
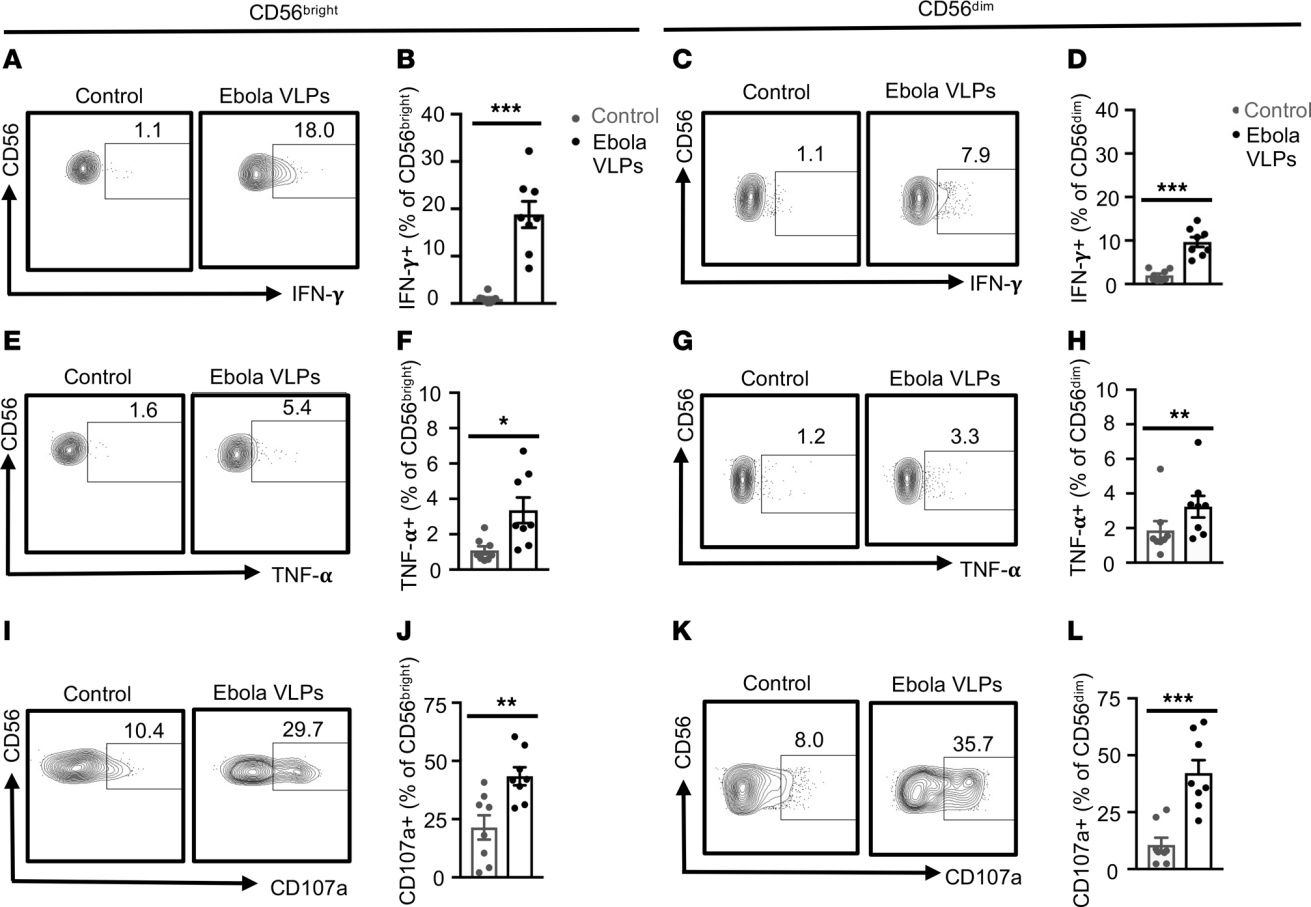
Both immature and mature NK cells respond to Ebola VLPs but to different degrees. PBMCs were stimulated with or without Ebola VLPs and stained as in Figures 2 and 3. Single and live CD3^–^CD56^+^ NK cells were divided into CD56^bright^ and CD56^dim^ NK cells as shown in Supplemental Figure 2. Representative dot blots and the corresponding summary data for IFN-γ (**A**–**D**), TNF-α (**E**–**H**), and CD107a (**I**–**L**) from 8 independent experiments with PBMCs from 8 donors are shown. Results are shown as mean ± SEM. *P < 0.05, **P < 0.01, ***P < 0.001 as calculated by 2-tailed paired Student’s *t* test.

NK cells can be separated into subpopulations based on their surface CD56 expression. More immature NK cells exhibit high CD56 expression and exhibit potent cytokine-secreting functionality (61–65). As NK cells mature, expression of CD56 is reduced concomitant with increased cytolytic function. We found that both CD56^bright^ and CD56^dim^ NK cells responded to the Ebola VLP stimulation (Figure 4). IFN-γ (Figure 4, A–D) and TNF- α (Figure 4, E–H) secretion was more pronounced in CD56^bright^ than in CD56^dim^ NK cells. Degranulation responses after Ebola VLP stimulation trended higher in CD56^dim^ than CD56^bright^ NK cells (Figure 4, I–L).

### EBOV VP40 is responsible for activation of NK cells.

**Figure 5:**
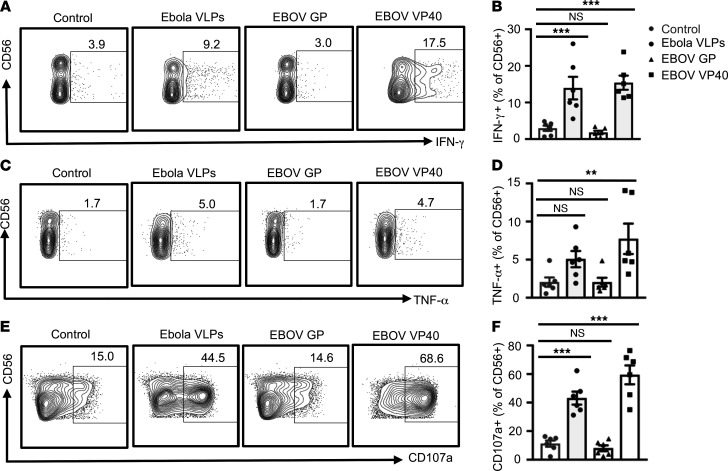
Activation of NK cells by Ebola VLPs is mediated through its EBOV VP40 component. PBMCs were left unstimulated or stimulated with Ebola VLPs (10 μg/mL), EBOV GP (10 μg/mL), or EBOV VP40 (5 μg/mL) for 24 hours (IFN-γ and TNF-α) or 48 hours (NK cell degranulation). Cells were stained for the surface and intracellular markers and data acquired as described earlier. Representative dot blots and the corresponding summary data showing gated CD3^–^CD56^+^ NK cells plotted for IFN-γ (**A** and **B**), TNF-α (**C** and **D**), and NK cells’ degranulation (CD107a surface expression) (**E** and **F**) from 6 independent experiments performed with PBMCs from 6 donors are shown. All results are shown as mean ± SEM. **P < 0.01, ***P < 0.001, as calculated by repeated measures (RM) 1-way ANOVA followed by Dunnett’s multiple comparisons test.

Past reports of NK cell stimulation in response to vesicular stomatitis virus– or adenovirus-based Ebola vaccines attributed this activity to EBOV GP (66–70). As our VLPs are composed of both EBOV GP and VP40 (Figure 1), we sought to delineate which of these 2 components contributed to the activation of human NK cells. We stimulated freshly isolated PBMCs with Ebola VLPs, purified recombinant EBOV GP (Supplemental Figure 4A, IBT Bioservices), or purified recombinant VP40 (Supplemental Figure 4B, MyBioSource). Similar to what was observed with the Ebola VLPs, stimulation of PBMCs with purified EBOV VP40 strongly induced expression of IFN-γ (Figure 5, A and B) and TNF-α (Figure 5, C and D) as well as degranulation (Figure 5, E and F) of NK cells. In most experiments, the observed effect of VP40 was slightly higher than that observed with Ebola VLPs. No stimulatory effect was observed following stimulation with purified recombinant EBOV GP (Figure 5). This observation was surprising and prompted us to investigate whether the native trimerized conformation of EBOV GP is necessary for its effect on NK cells. To answer this question, we stimulated PBMCs with equivalent amounts of VLPs bearing EBOV GP on EBOV VP40 core or those on HIV-1 Gag core (71) and studied NK cell activation status. Enhanced NK cell IFN-γ and TNF-α secretion and degranulation (CD107a surface expression) were observed only in NK cells that were exposed to VLPs bearing EBOV GP on EBOV VP40 core (Supplemental Figure 5, A–F). Functional EBOV GP has been reported to trigger IL-10 secretion in PBMC cultures (72). The fact that Ebola GP-HIV-1 Gag VLPs were able to induce 1L-10 secretion in our PBMC cultures (Supplemental Figure 5G) is evidence that the functional properties of EBOV GP are intact in VLPs with GP on HIV-1 Gag core. Together these observations strongly indicate that the observed stimulatory effect of Ebola VLPs on NK cell activation is mediated by monomeric and/or polymeric forms of EBOV VP40.

### Accessory cells are required for the EBOV VP40-induced optimal activation of NK cells.

**Figure 6:**
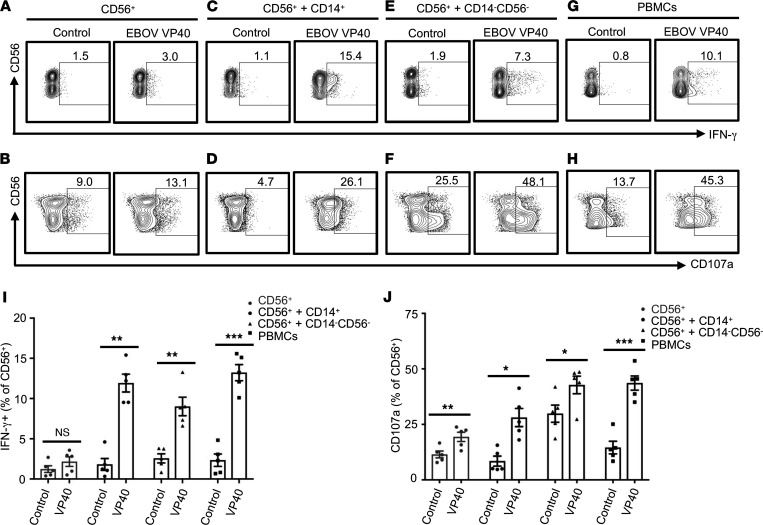
CD14^+^ and CD14^–^CD56^–^ accessory cells are required for the EBOV VP40-induced optimal NK cell activation. Purified CD56^+^ NK cells, cultured alone (**A** and **B**), with purified CD14^+^ cells (**C** and **D**), or with CD14^–^CD56^–^ fraction (**E** and **F**) and the parent PBMCs (**G** and **H**) were stimulated with or without EBOV VP40 (5 μg/mL) for 24 hours (IFN-γ) or 48 hours (CD107a). Cells were analyzed flow cytometrically. Representative dot blots with CD3^–^CD56^+^ NK cells plotted for IFN-γ (**A**, **C**, **E**, and **G**) and CD107a (**B**, **D**, **F**, and **H**) are shown. Corresponding summary data from 5 independent experiments performed with cells isolated from 5 donors are shown in **I** and **J**, respectively. Results are shown as mean ± SEM. *P < 0.05, **P < 0.01, ***P < 0.001, as calculated by 2-tailed paired Student’s *t* test.

In our initial attempts to recapitulate the Ebola VLP- or VP40-stimulated NK cell functional responses using purified CD56^+^ NK cells (Supplemental Figure 6A), we surprisingly observed diminished cytokine secretion and cytotoxicity responses of NK cells separated from the remaining cells in PBMCs (Figure 6, A and B). We hypothesized that accessory cells are likely involved in this effect, similar to what has been observed with influenza virus or other pathogen-derived stimuli (67, 73–75). To test this, we isolated autologous CD14^+^CD56^–^ myeloid and CD14^–^CD56^–^ nonmyeloid cells from the donor PBMCs (Supplemental Figure 6, B and C). CD56^+^ NK cells stimulated with EBOV VP40 in the presence of autologous CD14^+^ (Figure 6, C and D) or CD14^–^CD56^–^ cells (Figure 6, E and F), but not in the absence of these accessory cells, exhibited robust expression of IFN-γ (Figure 6, C and E) and degranulation responses (Figure 6, D and F) that were more similar to the response of NK cells in stimulated PBMCs (Figure 6, G and H) than those of isolated NK cells (Figure 6, A and B). These results bore out with cells from multiple donors (Figure 6, I and J) and when measuring TNF-α responses (Supplemental Figure 6, D–H). Unexpectedly, coculture of purified CD56^+^ cells with CD14^–^CD56^–^ accessory cells in the absence of EBOV VP40 stimulation resulted in elevated degranulation responses, which were further increased following EBOV VP40 stimulation (Figure 6, F and J). No such effect was observed on the cytokine secretions. Thus, CD14^+^ cells, and some yet-to-be-identified cell population in the CD14^–^CD56^–^ cellular fraction, serve as accessory cells in amplifying EBOV VP40-induced activation of NK cells. However, we do note that there was a trend toward higher frequencies of cytokine-secreting and degranulated NK cells in purified CD56^+^ cell cultures incubated with EBOV VP40 but without any additional cellular fraction (Figure 6, A, B, I, and J), suggesting that a subset of NK cells may directly respond to EBOV VP40.

Next, we tested whether the accessory cell–mediated EBOV VP40 effect on NK cell functions was mediated through soluble factors released by these cells upon activation or through cell-cell contact-dependent mechanism(s). To test these possibilities, we compared the effect of EBOV VP40-exposed accessory cells cultured with purified CD56^+^ cells in the presence or absence of Transwell inserts that block cell-cell contact. Enhancement of NK cell IFN-γ secretion and degranulation by accessory cells was partially dependent on cell-cell contact (Supplemental Figure 7). Nevertheless, some accessory cell–dependent increases in the responses of NK cells over those of purified NK cells persisted in the presence of Transwells, suggesting involvement of soluble factors as well (Supplemental Figure 7).

### Regulation of NK cell responses by EBOV VP40-induced cytokines.

**Figure 7:**
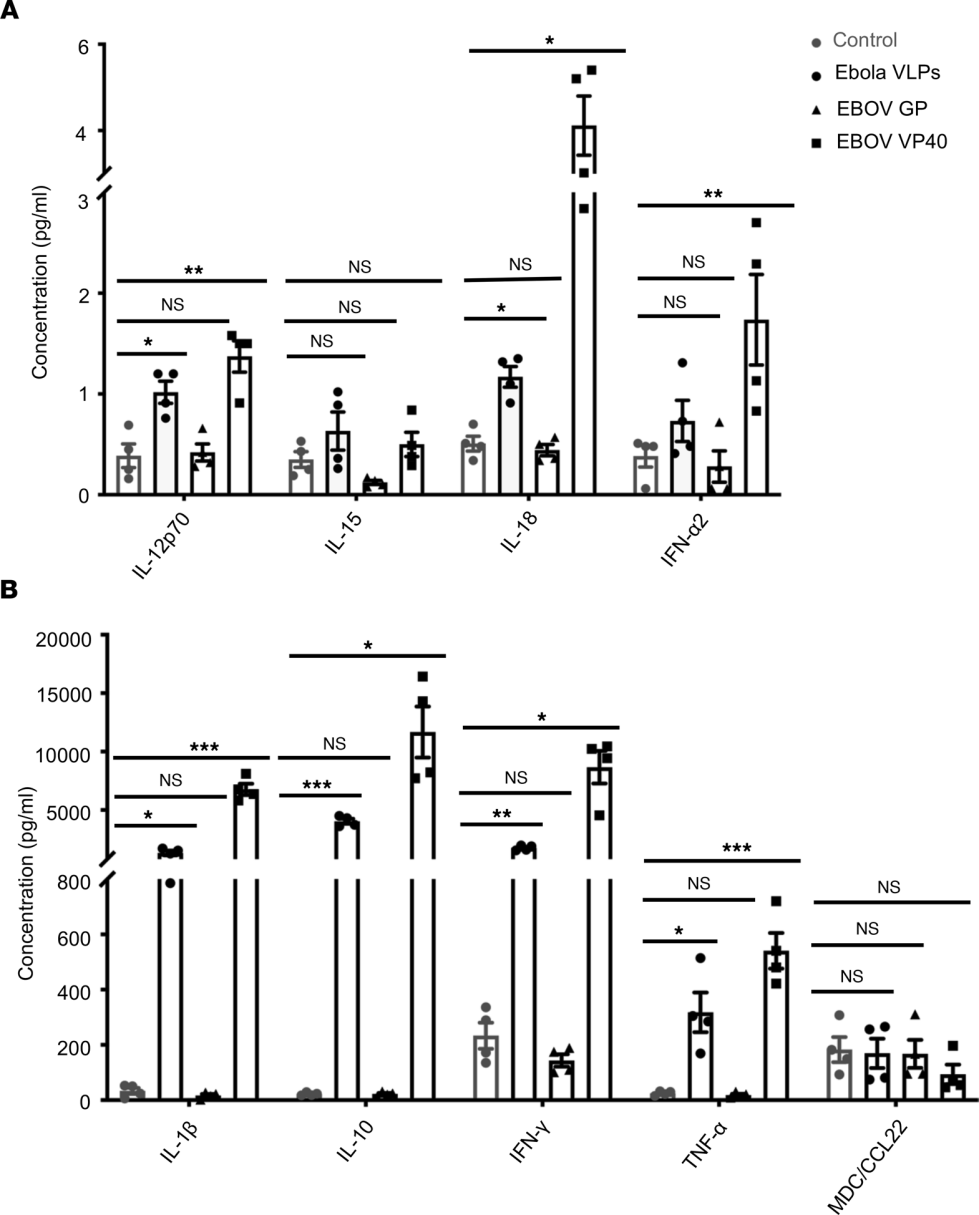
Secretion of inflammatory cytokines induced by Ebola VLPs and EBOV VP40. PBMCs were cultured in media alone (gray circles) or stimulated with Ebola VLPs (10 μg/mL, black circles), EBOV GP (10 μg/mL, triangles), or EBOV VP40 (5 μg/mL, squares) for 24 hours as described earlier. Amounts of IL-12p70, IL-15, IL-18, andIFN-α2 (**A**) and IL-1β, IL-10, IFN-γ, TNF-α, and MDC/CCL22 (**B**) secreted into the culture supernatants were quantified by using a Milliplex human cytokine/chemokine multiplex kit. Results are shown as mean ± SEM of data from 4 donors. *P < 0.05, **P < 0.01, ***P < 0.001, as calculated by RM 1-way ANOVA followed by Dunnett’s multiple comparisons test. MDC, macrophage-derived chemokine.

NK cell responses can be regulated through the action of various cytokines secreted as a result of the activation of various cells responding to pathogen-derived stimuli (67, 73–75). Using a custom-designed Luminex assay, we found that IL-12p70, IL-18, IFN-α2, IL-1β, IL-10, IFN-γ, and TNF-α were measurably secreted following stimulation of PBMCs with Ebola VLPs or EBOV VP40 but not EBOV GP (Figure 7). Expression of IL-15 and MDC/CCL22 was unchanged between control and stimulated cultures. Together, these findings indicate that Ebola VLP-induced activation of accessory cells results in cytokine secretion that partly triggers the optimal activation of NK cells.

**Figure 8:**
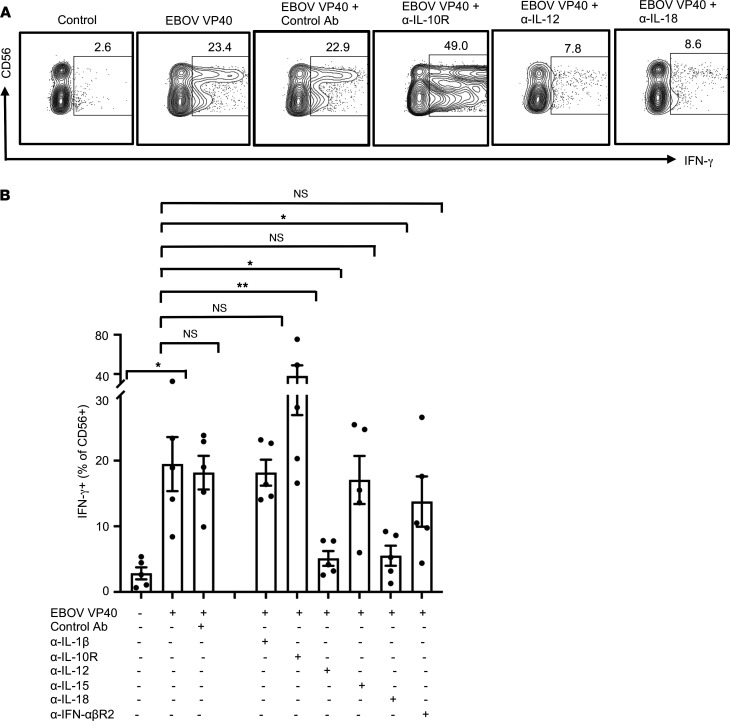
IL-12 and IL-18 regulate EBOV VP40-induced NK cell IFN-γ responses. PBMCs were cultured in complete media unstimulated or stimulated with EBOV VP40 (5 μg/mL) for 24 hours. Where indicated blocking monoclonal antibodies for IL-1β, IL-10R, IL-12, IL-15, IL-18, IFN-αβR2 or the corresponding isotype controls were included in the cultures (final concentration of 3 μg/mL). Cells were analyzed flow cytometrically as described earlier. Representative dot blots for CD3^–^CD56^+^ NK cells secreting IFN-γ in the presence of the blocking antibodies that showed statistically significant effect compared with the EBOV VP40 alone are shown (**A**). Corresponding summary data from 5 independent experiments performed with PBMCs from 5 donors are shown (**B**). Results are shown as mean ± SEM. *P < 0.05, **P < 0.01, as calculated by RM 1-way ANOVA followed by Holm-Šidák multiple comparisons test. IL-10R, IL-10 receptor; IFN-αβR2, IFN-αβ receptor 2.

**Figure 9:**
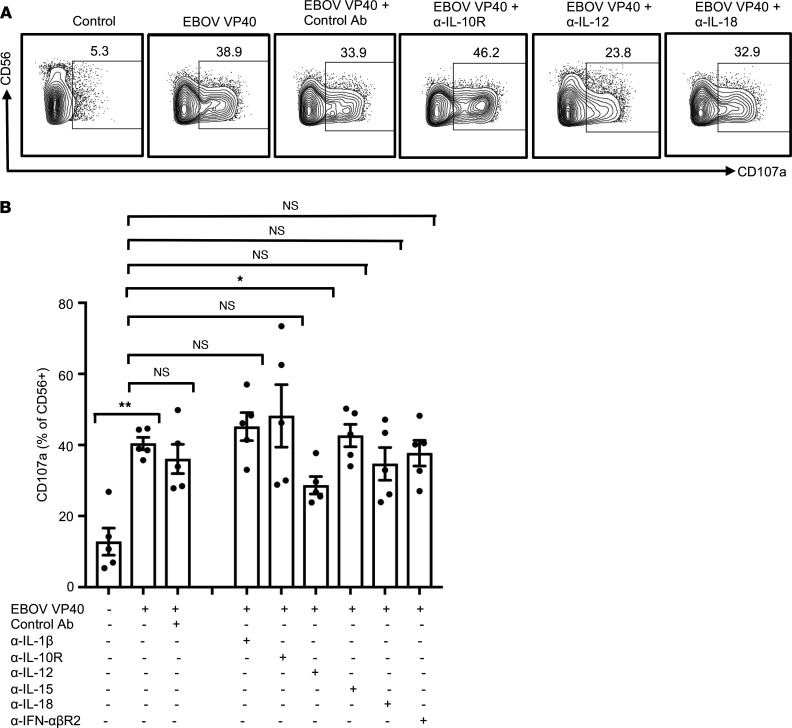
IL-12 regulates EBOV VP40-induced NK cell degranulation. PBMCs were cultured, then stimulated with EBOV VP40 (5 μg/mL) for 48 hours in the presence of blocking monoclonal antibodies for IL-1β, IL-10R, IL-12, IL-15, IL-18, and IFN-αβR2 or the corresponding isotype controls (all at a final concentration of 3 μg/mL) and analyzed flow cytometrically as described earlier. Representative dot blots with CD3^–^CD56^+^ NK cells plotted for surface CD107a are shown (**A**). Corresponding summary data from 5 independent experiments performed with PBMCs from 5 donors are shown (**B**). Results are shown as mean ± SEM. *P < 0.05, **P < 0.01, as calculated by RM 1-way ANOVA followed by Holm-Šidák multiple comparisons test.

To define the role of these cytokines in EBOV VP40-stimulated NK cell activation, we used monoclonal antibodies capable of blocking each of these cytokines individually. Blockade of either IL-12 or IL-18 significantly impaired the expression of IFN-γ (Figure 8, A and B) and TNF-α (Supplemental Figure 8, A and B) by NK cells in VP40-stimulated PBMCs. Little effect was observed with IL-1β, IL-15, or IFN-αβR2 blockade. In contrast, IL-10R blockade significantly enhanced cytokine production by NK cells in VP40-stimulated cultures (Figure 8, A and B; and Supplemental Figure 8, A and B). In contrast to cytokine production, blockade of IL-12, but not that of IL-10 or IL-18, significantly inhibited VP40-induced degranulation responses of NK cells (Figure 9, A and B). These results suggest different but overlapping mechanisms involved in EBOV VP40-stimulated activation of NK cell cytokine secretion and cytotoxicity responses.

## Discussion

In this study we utilized Ebola VLPs consisting of EBOV VP40 core and EBOV GP spikes as surrogates to study the innate immune behavior of NK cells when they first encounter Ebola virus or its antigens. Exposure of healthy human PBMCs to Ebola VLPs or VP40, but not GP, enhanced the expression of cytokines and cytolytic mediators by NK cells and increased their cytotoxic functionality. This NK cell response to VP40 required the presence of CD14^+^ monocytes/macrophages and other less-defined cells as the accessory cells. IL-12 and IL-18 were important for the stimulation of cytokine production, whereas IL-12 was required for enhanced cytolytic activity. Moreover, VP40-stimulated IL-10 could constrain the resulting cytokine production by NK cells. These results provide potentially novel mechanistic insights into activation of human NK cells in response to Ebola virus gene products.

Data from fatal human cases of EVD and those from animal models suggest a generalized dysregulation of innate immune responses during the early stages of Ebola virus infections (76–78). The actions of certain Ebola virus proteins, including VP24 and VP35, result in the functional impairment of macrophages and dendritic cells, generating a cytokine storm and thereby preventing effective crosstalk between the innate and adaptive arms of the immune system that is necessary for mounting an effective adaptive immune response. This innate immune malfunctioning results in uncontrolled virus replication and systemic dissemination that can overwhelm host defenses (22, 44–46, 76, 78–80). Though substantial data on the role of monocytes/macrophages, dendritic cells, B cells, and T cells during the course of Ebola infection have been collected in recent years, the fate of NK cells is not clear, except that their peripheral numbers decline because of massive lymphocyte apoptosis (47). In a mouse model of EVD, the decrease in peripheral NK cells was observed concomitant with their accumulation in organs like the liver, where they contribute to the exacerbation of the disease through their cytotoxic activities (52). This suggests that during Ebola virus infection certain viral proteins may stimulate the activation of NK cells, which in turn damage the virally infected tissues. These activated tissue NK cells may also eliminate the effector lymphocytes that infiltrate into the infected tissues thereby preventing the elimination of virally infected cells and further contributing to the tissue damage.

On the other hand, prophylactic immunization with Ebola VLPs has been shown to provide protection against subsequent challenge with lethal doses of Ebola viruses (53–56). Surprisingly, in one such study, Ebola VLP–immunized animals were protected against a subsequent lethal Ebola virus challenge merely a day after the immunization, strongly suggesting that the protection was likely mediated through the cells of the innate immune system. Perforin-mediated cytotoxicity of NK cells was absolutely essential for this protection as abrogation of NK cells or perforin led to the loss of the observed protection. The fact that EBOV GP-containing VLPs as well as those lacking it but retaining VP40 core were equally capable of inducing protection strongly indicated that VP40 was integral to this protection (56). This protection was mediated through the rapid elimination of Ebola virus–infected cells by the VLP-activated NK cells thereby limiting the virus spread. In another study, administration of Ebola VLPs 24 hours after infection with Ebola virus protected the mice from EVD. This protection required perforin, B cells, T cells, macrophages, and dendritic cells, though NK cells were dispensable. The protection was GP-dependent as VLPs lacking GP were not protective (81).

Given these divergent contexts, our data reveal that Ebola VP40 can enhance the cytolytic and cytokine-producing functionalities of human NK cells. Based on their CD56 surface expression, NK cells separate into CD56^bright^ and CD56^dim^ subpopulations that correspond to immature and mature NK cells, respectively. Upon activation, CD56^bright^ NK cells respond mainly by secreting cytokines, like IFN-γ, whereas mature CD56^dim^ NK cells release contents of their cytotoxic granules that, in turn, lyse the target cells (61–65). In line with this, though both CD56^bright^ and CD56^dim^ NK cell subpopulations responded to Ebola VLPs, cytokine responses were more pronounced in CD56^bright^ NK cells while there was a trend toward higher degranulation in CD56^dim^ NK cells. The separation of NK cells into CD56^bright^ and CD56^dim^ subpopulations started diminishing 24 hours after stimulation with Ebola VLPs, suggesting activation induced changes in CD56 expression and/or NK cell differentiation. Our findings thus suggest distinct but overlapping mechanisms involved in these 2 Ebola VLP-activated NK cell functionalities. The stimulatory capacity of VLPs required overnight incubation with PBMCs, reinforcing the necessity of uptake of VLPs and subsequent cytokine production by intermediate accessory cells. Ebola VLP-induced stimulation of NK cells was mediated through its VP40 component. We did not observe any measurable NK cell cytokine secretion or degranulation after stimulation of PBMCs with purified Ebola GP. This observation is in agreement with a previous study showing lack of Ebola GP-induced NK cell activation in freshly isolated PBMCs from nonvaccinated human donors cultured in media containing prevaccination sera collected from volunteers participating in a heterologous Ad26 ZEBOV and Modified Vaccinia Ankara–MVA-BN-Filo (MVA-BN-Filo) vaccine trial. Measurable NK cell activation was detected only in PBMCs cultured in the presence of postvaccination sera, highlighting the importance of anti-Ebola GP antibodies in GP-induced NK cell activation (68). On the other hand, our findings are partially distinct from those of Wagstaffe et al. (67), who showed Ebola GP-induced NK cell degranulation, but lack of IFN-γ secretion, in frozen PBMCs collected from vaccinees receiving the 2-dose Ad26 ZEBOV and MVA-BN-Filo vaccine regimen. The observed difference could be because of the different culture conditions employed in the 2 studies. Whereas we used freshly isolated PBMCs from healthy donors, cultured them in media containing normal human serum, and stimulated them for 48 hours with Ebola GP lacking its transmembrane domain, Wagstaffe et al. (67) used frozen PBMCs from vaccinees, cultured in media containing autologous sera and stimulated for only 8 hours with a full-length version of Ebola GP. However, our data showing the lack of NK cell cytokine secretion and surface CD107a upregulation upon stimulation with the VLPs containing full-length GP but expressed on HIV-1 Gag core strongly suggest that the observed difference is not because of the lack of the GP transmembrane domain. Functional integrity of Ebola GP in VLPs on HIV-1 Gag core is further highlighted by its ability to induce IL-10 (72). Our finding that EBOV VP40 exposure alone can stimulate NK cells in freshly isolated PBMCs is in agreement with the activated NK cell–dependent protection observed in animals vaccinated with Ebola VLPs consisting of VP40 core but lacking GP spikes (56). How VP40, forming the core of Ebola VLPs, is able to stimulate NK cells is intriguing, though. Different possibilities include some VP40 molecules being exposed on the VLP envelope, or perhaps VP40 coming from incompletely enveloped VLPs. Alternatively, VLPs could be engulfed by the accessory cells and processed, and the VP40 released in the cytoplasm could then mediate its effect by activating those accessory cells. The fact that only slight Ebola VLP-elicited stimulation was observed in purified CD56^+^ cells further points to this mechanism.

Because of their known ability to modulate the functions of NK cells through the secretion of cytokines and other factors, activated CD14^+^ monocytes/macrophages were thought to be the likely accessory cells (67, 73). This turned out to be true as we were able to recapitulate the Ebola VLP effect on NK cells upon mixing autologous CD56^+^ and CD14^+^ cells. Our data showing lower degree of NK cell degranulation in Ebola VLP–stimulated autologous CD56^+^ and CD14^+^ cell cultures than that observed in the corresponding stimulated PBMC cultures led us to discover that cells other than monocytes/macrophages are also playing some kind of accessory role. This effect appeared to be more cell-cell contact dependent, though soluble mediators also appeared to play some role. Two additional observations were made from these data. First, in most experiments, a small percentage of purified CD56^+^ cells responded to Ebola VLPs or purified EBOV VP40 even in the absence of CD14^+^ or any other cells. This could be because of the residual number of cellular contaminants present in our isolated CD56^+^ cells. At this time, we have not ruled out the possibility that a small subset of NK cells is able to respond to EBOV VP40 directly. Further experiments are needed to explore this possibility. Second, mixing of CD56^+^ and CD14^+^ cells together, in the absence of any stimulus, marginally decreased basal NK cell degranulation while mixing of CD56^+^ and CD14^–^CD56^–^ cells increased this. This is possibly due to the secretion of suppressive cytokine(s), such as IL-10, by the monocytes/macrophages. Their absence in the CD56^+^ and CD14^–^CD56^–^ cell cocultures likely led to the increased basal NK cell degranulation. Nonetheless, stimulation with Ebola VLPs or VP40 was sufficient not only to overcome this suppression but also to induce additional activation of the NK cells.

Detection of enhanced secretion of cytokines including IL-12, IL-18, IFN-α2, IL-1β, and IL-10 upon incubation of primary human PBMCs with Ebola VLPs or VP40 suggested that activated CD14^+^ monocytes/macrophages and/or other accessory cells mediate the observed effect through one or more of these cytokines. It is interesting to note here that levels of some of these cytokines, especially IL-1β, are also highly elevated during the early stages of EVD (82). Secretion of both activating cytokines, like IL-12 and IL-18, and suppressive cytokine IL-10 upon Ebola VLP stimulation suggest the generation of a complex environment of NK cell–activating and –suppressive factors. The net balance of the signals generated through the action of these factors likely determines whether an NK cell becomes activated or not. This hypothesis is supported by our data that revealed that blocking of IL-12 and IL-18 in the cultures significantly reversed the Ebola VLP effect on NK cell cytokine secretions while IL-10R blockade exacerbated this effect (Figure 8). The rapid production of IL-10 in response to infections is well documented in preventing uncontrolled cytokine storm (83), and the resulting immune-suppressive effects on NK cells may prevent their activation. This may explain why the observed level of IL-10 alone does not correlate well with protection from EVD (80). However, we show that Ebola VLP-stimulated secretion of IL-12 and IL-18 by CD14^+^ cells and/or other accessory cells can overcome the suppressive effect of IL-10 and together can provide strong activating signals, shifting the net balance toward NK cell activation. In contrast to the cytokine secretion, only IL-12 blockade reversed the Ebola VLP or VP40-induced NK cell degranulation, while IL-18 and IL-10R blockade showed no significant effect. This is in line with different kinetics of NK cell cytokine secretion and degranulation and further support the idea of different but overlapping mechanisms involved in cytokine secretion and cytotoxic granule release upon activation of NK cells (6, 84).

Together these findings contribute to our understanding of NK cell behavior on exposure to Ebola virus and/or its proteins and may help in devising better countermeasures against EVD. Potential therapeutic regimens that include agents that modulate NK cell activity may be helpful in arresting or managing the devastating consequences of EVD during the early stages of the disease. From the point of view of ring vaccination strategies, to protect healthcare workers and contain Ebola outbreaks, or bioterrorism event(s), inclusion of EBOV VP40 in Ebola vaccines has the potential to add acute elevation of NK cell functionality as a component of rapid protection against Ebola viruses. The ability of VP40-activated NK cells to eliminate Ebola-infected cells and limit viral spread may enable sufficient time for protective, adaptive, antiviral immune responses to develop.

## Methods

### Plasmids and generation of EBOV GP-VP40 VLP production cell line.

Supplemental Figure 1 shows the schematics of 2 plasmids that have been used in this study. Plasmid pcDNA5/TO-puro *EBOV GP* (Mayinga) was created by placing a codon-optimized *EBOV GP* (Mayinga) gene (accession AAC54887.1) under a tetracycline-controlled cytomegalovirus promoter as described earlier (60, 71). Plasmid pcDNA4/TO-zeo *EBOV VP40* was created by placing codon-optimized *EBOV VP40* (Makona-Kissidougou-C15) gene (accession KJ660346.1) into pcDNA4/TO-zeo plasmid by using KpnI and XbaI sites. Protein expression of EBOV GP and VP40 was tested by transient transfections of 293T cells (Thermo Fisher Scientific) cultured in complete DMEM: DMEM (Gibco) supplemented with Glutamax (Gibco), 100 IU/mL penicillin and 100 μg/mL streptomycin (both Mediatech), and 10% fetal bovine serum (FBS) (MilliporeSigma). Briefly, 293T cells, at approximately 70% confluence, were transfected with individual plasmids using Lipofectamine 2000 (Invitrogen). Twenty-four hours later cells were harvested and washed, and cell lysates were prepared and resolved on SDS-PAGE gels (Invitrogen) followed by protein transfer onto the nitrocellulose membranes (MilliporeSigma). Membranes were blocked, then incubated overnight with specific anti-EBOV GP (catalog 0301-015, IBT Bioservices) or anti-EBOV VP40 antibodies (catalog 0301-010, IBT Bioservices), followed by another incubation with the IRDye-conjugated secondary antibodies (catalog 926-32211, LI-COR Biosciences). Membranes were thoroughly washed, and images were captured on Odyssey CLx imaging system (LI-COR Biosciences).

The 293F 6T/R cell line (Thermo Fisher Scientific) was used to generate a stably transfected and inducible cell line secreting EBOV GP-VP40 VLPs. Briefly, cells were cultured in FreeStyle 293 Expression media (Thermo Fisher Scientific) supplemented with 5.0 μg/mL blasticidin (InvivoGen) to a cell density of approximately 1.0 × 10^6^ cells/mL and transfected with pcDNA5/TO-puro *EBOV GP* and pcDNA4/TO-zeo *EBOV VP40* plasmids. Forty-eight hours later, cells were shifted into 293 Expression media supplemented with 5.0 μg/mL blasticidin, 1.0 μg/mL puromycin, and 10.0 μg/mL zeocin (both from InvivoGen), and antibiotic-resistant cells were allowed to expand over the next 4 weeks with periodic changes with antibiotic-containing fresh media. Cells so selected were either induced with 2.0 μg/mL doxycycline (MilliporeSigma) or left as such. Twenty-four hours later cells were harvested, and cell lysates were probed by Western blotting using specific antibodies against EBOV GP, EBOV VP40, and β-actin (clone C4, Santa Cruz Biotechnology). Cells showing high expression of inducible EBOV GP and VP40 were labeled as stable EBOV GP-VP40 293F cells and used for the subsequent studies.

### Production and characterization of Ebola VLPs.

EBOV GP-VP40 293F cells were suspended in 293 Expression media supplemented with blasticidin, puromycin, and zeocin in 500 mL or 1 L Erlenmeyer flasks and allowed to expand on a shaker set at 125 rpm, inside a 37°C, 8% CO_2_ incubator. Upon reaching a cell density of approximately 1.0 × 10^6^/mL, cultures were induced with 2.0 μg/mL of doxycycline for 40 hours. Culture supernatants were harvested by centrifuging the cultures at 5000*g* for 10 minutes at 10°C in a high-speed cold centrifuge. Supernatants were further cleared by passing through a sterile filtration unit with a pore size of 0.45 μm. VLPs in the cleared supernatants were isolated by ultracentrifugation. Briefly, supernatants were layered onto sterile 20% sucrose cushions and tubes centrifuged at 30,000 rpm (110,500*g*) at 4°C for 1.5 hours in an ultracentrifuge (SW32 Ti rotor, Beckman Coulter). After centrifugation, supernatant was removed carefully, and Ebola VLP pellets left at the bottom of the tubes were resuspended in sterile cold phosphate-buffered saline (PBS). VLPs and the corresponding cell lysates were probed for EBOV GP and EBOV VP40 by Western blotting as described above. Buoyant density of Ebola VLPs was determined by layering the VLPs isolated as described above, on the top of 20% to 60% sucrose density gradient and centrifuging the tubes at 35,000 rpm (151,200*g*) at 4°C for 16 hours (SW41 rotor, Beckman Coulter). Starting at the top, 12 equal fractions were collected carefully. A small portion of each fraction was used to probe for EBOV GP and EBOV VP40 by Western blotting while the remainder was used to measure its refractive index on a refractometer. Buoyant density of each fraction was calculated, and the fraction(s) maximally enriched for EBOV GP and EBOV VP40 were identified. All Ebola VLP, recombinant Ebola GP, and VP40 lots used in this study were pretested for the presence of endotoxin by using a chromogenic LAL Endotoxin Assay Kit (GenScript), as per manufacturer’s instructions. Endotoxin presence in different lots was extremely low and varied between 0.43 and 0.97 EU/mL for Ebola GP-VP40 VLP, 0.47 and 0.99 EU/mL for Ebola GP-HIV-1 Gag VLP, 0.84 and 1.00 EU/mL for recombinant EBOV GP, and 0.024 and 0.51 EU/mL for recombinant EBOV VP40. These endotoxin levels were comparable to those in complete media prepared with the certified low-endotoxin human serum used in our studies. The fact that NK cell activation was observed only upon stimulation with Ebola GP-VP40 VLP and VP40, and not with Ebola GP-HIV Gag VLP and GP that had comparably low endotoxin levels, shows that the observed effects on NK cells are Ebola GP-VP40 VLP and VP40-specific.

### Electron microscopy.

Shape, size, and structure of Ebola VLPs were studied by negative electron microscopy. Briefly, isolated VLPs resuspended in cold PBS were dropped on Nickel grids of 300 mesh with Formvar-supporting membranes (Electron Microscopy Sciences) that were glow discharged just before use. After cleaning, grids were air-dried and subjected to negative staining with 1% phospho-tungstic acid. Cleaned and air-dried grids were examined under a Hitachi H-7650 Transmission Electron Microscope (Hitachi High-Technologies) at Cincinnati Children’s Pathology Research Core.

### Isolation of human PBMCs, CD56^+^ NK cells, CD14^+^ monocytes, and CD14^–^CD56^–^ cells (negative fraction).

PBMCs were isolated from healthy human donors’ blood (provided by Cell Processing Core, Cincinnati Children’s Hospital Medical Center). Briefly, blood was collected in BD Vacutainer cell preparation tubes with sodium citrate (Thermo Fisher Scientific), and tubes were spun at 1600*g* for 30 minutes at room temperature, without break. Cells were collected and any residual red blood cells, if present, lysed and remaining cells washed thoroughly with PBS. PBMCs so obtained were either resuspended in complete RPMI medium (RPMI 1640, Thermo Fisher Scientific) supplemented with 10% heat-inactivated human AB serum (Thermo Fisher Scientific) or FBS as appropriate, 1× Glutamax, 100 IU/mL penicillin, and 100 μg/mL streptomycin, with 200 IU/mL of human recombinant interleukin-2 (rIL-2) (R&D Systems) at a cell density of 1 × 10^6^ cells/mL and used in the downstream experiments or were cryopreserved in freezing media (90% human serum + 10% DMSO) for future use. For CD56^+^ NK cell isolation, PBMCs were resuspended in sorting buffer (PBS, 0.5% BSA, 2 mM EDTA) at a cell density of 10^7^ cells/40 μL and CD56^+^ cells were isolated by positive selection over a magnetic column by using MACS CD56 microbeads as per the manufacturer’s instructions (Miltenyi Biotech, GmbH). Flow-through fraction from this isolation was used to isolate CD14^+^ monocytes by negative selection using human pan-monocyte isolation kit (Miltenyi Biotec, GmbH). Bound fraction from this step was eluted and labeled as CD14^–^CD56^–^ cells (negative fraction). Isolated CD56^+^ NK cells, CD14^+^ monocytes, and CD14^–^CD56^–^ cells were resuspended in complete RPMI medium supplemented with 200 IU/mL of rIL-2 at a cell density of 1 × 10^6^ cells/mL and used in the downstream experiments. Purity of the isolated cell populations was assessed by flow cytometry using APC-conjugated mouse anti-human CD56 (clone CMSSB, MBL International) and V500-conjugated mouse anti-human CD14 (clone M5E2, BD Biosciences) antibodies. In some experiments, freshly isolated PBMCs were labeled with PE-Cy7–conjugated mouse anti-human CD16 (clone 3G8, BD) and APC-conjugated mouse anti-human CD56 antibodies, and CD16^+^CD56^+^ cells were sorted aseptically on a BD FACSAria sorter.

### Cell activations.

PBMCs, isolated CD56^+^ NK cells, CD14^+^ monocytes, CD14^–^CD56^–^ cells, either alone or in combination (CD56^+^:CD14^+^ = 1:1, CD56^+^:CD14^–^CD56^–^ = 1:2), were resuspended in complete RPMI containing rIL-2, at a cell density of 1 × 10^6^ to 2 × 10^6^ cells/mL. Where appropriate, Alexa Fluor 700–conjugated mouse anti-human CD107a (clone H4A3, BD) antibody was added to the cells, and cells were transferred to 24-well tissue culture plates at 0.5 mL/well. Cells were stimulated with 0.5 mL of different stimuli (at final concentration of Ebola GP-VP40 VLPs: 10 μg of EBOV GP/mL, Ebola GP-HIV-1 Gag VLPs: 10 μg of EBOV GP/mL, recombinant EBOV GP [IBT Bioservices]: 10 μg/mL, recombinant EBOV VP40 [MyBioSource, Inc.]: 5 μg/mL). Optimal Ebola GP-VP40 VLP concentration used in this study was determined after initial experiments using a range of Ebola VLP concentrations. Recombinant Ebola GP and VP40 concentrations were selected based on the respective measured concentrations of each component within Ebola VLPs so that quantities of each protein were equal to that provided by stimulation with VLPs. Identical or comparable Ebola GP and VP40 concentrations have been used by other groups to study various immune cell functionalities (67–70, 85–87). Cells with media alone were included as the negative controls. Where indicated, the following blocking antibodies or the corresponding isotype controls were included during the activation at a final concentration of 3 μg/mL: anti–hIL-1β (clone 4H5, InvivoGen); anti–IL-10R (clone 3F9, BioLegend); anti–IL-12 p70 (clone 20C2, BD); anti–IL-15 (clone ct2nu, Thermo Fisher Scientific); anti–IL-18 (clone 125-2H, MBL International); anti–IFN-αβR2 (clone MMHAR-2, MilliporeSigma); and mouse IgG1 (clone P3.6.2.8.1), mouse IgG2a (clone eBM2a), rat IgG1 (clone eBRG1), and rat IgG2a (clone eBR2a) isotype controls (all from Thermo Fisher Scientific). Plates were incubated in a 37°C, 5% CO_2_ incubator for indicated lengths of time. Six hours before harvesting, a mix of GolgiPlug and GolgiStop (both from BD) was added to each well. Cells were harvested, washed thoroughly, and analyzed by flow cytometry. In the experiments conducted using the Transwell plates (Corning), CD56^+^ NK cells were added to the bottom of the wells while stimulated CD14^+^ or CD14^–^CD56^–^ cells were added on the top of the filter membrane thus physically separating them.

### Flow cytometry.

Cells were transferred to the FACS tubes, washed, and stained with Live/Dead fixable violet cell stain (Thermo Fisher Scientific). After washing, 5 μL of human TruStain FcX (BioLegend) was added to each tube, and cells were stained with anti-human CD3-BV605 (clone SP34-2, BD) and anti-human CD56-APC (clone CMSSB, MBL International) antibodies. Cells were washed, fixed, and permeabilized using Fixation/Permeabilization buffer set (BD). Cells in each tube were divided into 2 new tubes. One set was stained with mouse anti-human IFN-γ–FITC (clone 4S.B3, BioLegend) and mouse anti-human TNF-α–APC Cy7 (clone Mab11, BioLegend) antibodies while the other was stained with mouse anti-human granzyme B–FITC (clone GB11, BioLegend) and mouse anti-human perforin-PE (clone B-D48, BioLegend) antibodies. Cells were washed and data acquired on BD Fortessa and analyzed using FlowJo software (Tree Star, Inc.).

### Luminex assay.

PBMCs were left unstimulated or stimulated for 24 hours with Ebola VLPs, EBOV GP, or EBOV VP40 as described earlier. Culture supernatants were collected, cleared of any debris by centrifugation at 5000*g* for 15 minutes at 4°C,and tested for IL-1β, IL-10, IFN-γ, TNF-α, MDC/CCL22, IL-12p70, IL-15, IL-18, and IFN-α2 by using a Milliplex human cytokine/chemokine/growth factor panel multiplex kit (MilliporeSigma), as per manufacturer’s instructions. Briefly, 25 μL of sample supernatant was incubated with 25 μL of antibody-coated beads in a 96-well black plate overnight at 4°C on a continuous plate shaker. The next morning, plates were washed twice using the BioTek 405 TS, and 25 μL of secondary antibody was added to each well. Plates were incubated at room temperature for 1 hour while shaking. Finally, 25 μL/well of streptavidin-RPE was added directly to the secondary antibody, and plates were incubated for another 30 minutes at room temperature while shaking. Following incubation, plates were washed twice, and 150 μL of sheath fluid was added to each well. Plates were kept shaking for 5 minutes and data acquired using Luminex technology on a Milliplex Analyzer (MilliporeSigma). Absolute concentrations of various cytokines/chemokines/growth factors were calculated from the respective standard curves using Bio-Plex Manager 6.2 software (Bio-Rad Laboratories).

### Cytotoxicity assay.

An Ebola ADCC assay previously developed in our laboratory (60) was used to assess the cytotoxic potential of Ebola VLP-exposed cells. Briefly, 293 Trex cells (target cells), stably transfected with luciferase and *EBOV GP* genes, were induced with 2.0 μg/mL of doxycycline for 24 hours. Cells were harvested, washed, and mixed with PBMCs (effector cells), either unexposed (control) or those exposed to Ebola VLPs for 48 hours, at different effector/target cell ratios (10:1, 5:1, and 2:1) in complete media in 96-well plates. All the wells received 1:200 diluted rhesus macaque anti-EBOV GP plasma (71). Plates were incubated at 37°C, 6% CO_2_ for 6 hours. Following incubation, cells were washed thrice with PBS and cell pellets resuspended in 100 μL/well of complete media. Cells were lysed with the addition of 100 μL/well of Britelite Plus luciferase substrate (PerkinElmer), and luciferase activity (relative luciferase units, RLU) was measured on a BioTek Synergy HTX reader. Percentage target cell killing was calculated by the following formula: ([RLU_control_ – RLU_anti-EBOV GP plasma_] × 100)/RLU_control_.

### Statistics.

Statistical analysis was performed using 2-tailed Student’s *t* test, or RM 1-way ANOVA followed by a multiple comparisons test, and using GraphPad Prism software version 8. A *P* < 0.05 was considered significant.

### Study approval.

This study did not involve use of any laboratory animals or direct participation of human volunteers. The study was approved by Cincinnati Children’s Hospital Medical Center’s Institute Biosafety Committee. Blood used in this study was provided by Cell Processing Core, Cincinnati Children’s Hospital Medical Center.

## Author contributions

HL conducted experiments and acquired data; PS and SNW contributed to interpreting the data and writing the manuscript; and KS designed research studies, supervised all experiments and data collection, analyzed the data, and wrote the manuscript.

## Supplementary Material

Supplemental data

## Version 1 07/21/2022

In-Press Preview

## Version 2 08/22/2022

Electronic publication
