# Supplementary material for: Ebola virus protein VP40 stimulates IL-12– and IL-18–dependent activation of human natural killer cells
Source: JCI Insight. 2022 Aug 22;7(16):e158902. doi: 10.1172/jci.insight.158902 (PMC9462474; doi:10.1172/jci.insight.158902)
Supplement: Supplemental data [file jciinsight-7-158902-s214.pdf]

Supplemental Material

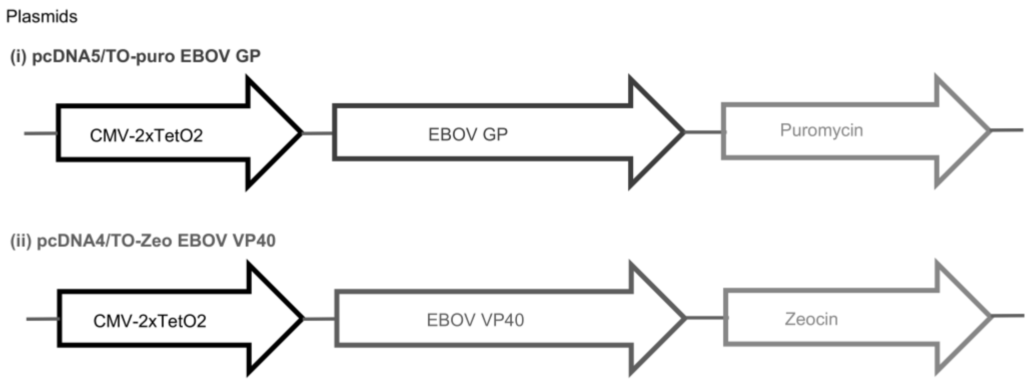

Supplemental Figure S1. Plasmids used in the current study to generate Ebola VLPs. Schematics of (i) pcDNA5/TO-puro EBOV GP and (ii) pcDNA4/TO-zeo EBOV VP40 plasmids used to develop EBOV GP-VP40 293F stable cell line.

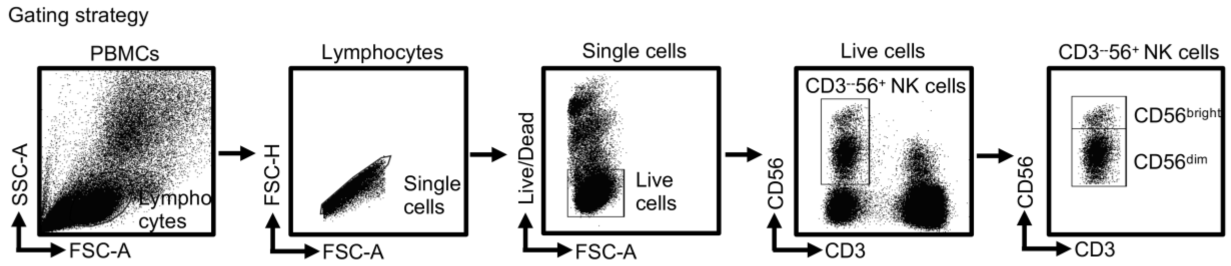

Supplemental Figure S2. Strategy for gating $CD3^-CD56^+$ NK cells and $CD56^{bright}$ or $CD56^{dim}$ subsets of NK cells in PBMCs.

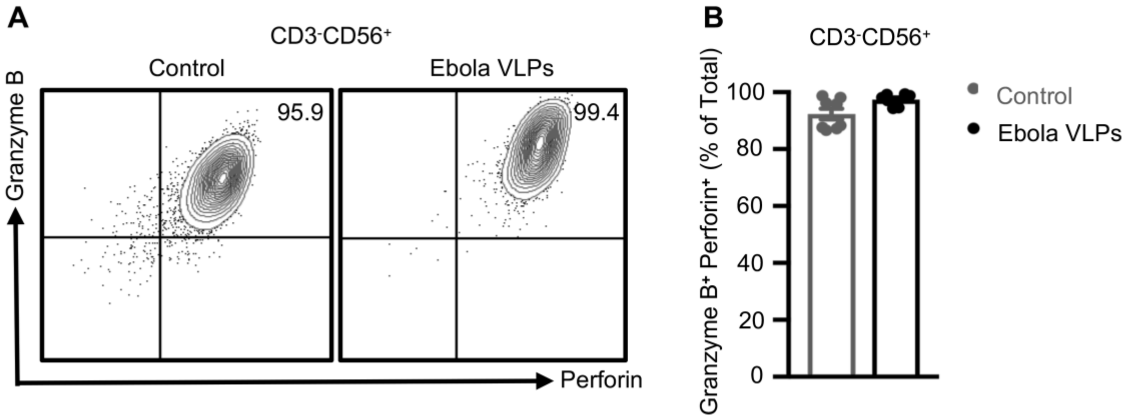

Supplemental Figure S3. Frequencies of granzyme B and perforin positive NK cells after stimulation with Ebola VLPs. PBMCs were stimulated with or without Ebola VLPs for 48 hours. Dot blots for $CD3^{-}CD56^{+}$ NK cells stained for intracellular granzyme B and perforin are shown in (A). (B) Corresponding summary data showing mean ± SEM of values obtained from 8 independent experiments performed with PBMCs from 8 different donors.

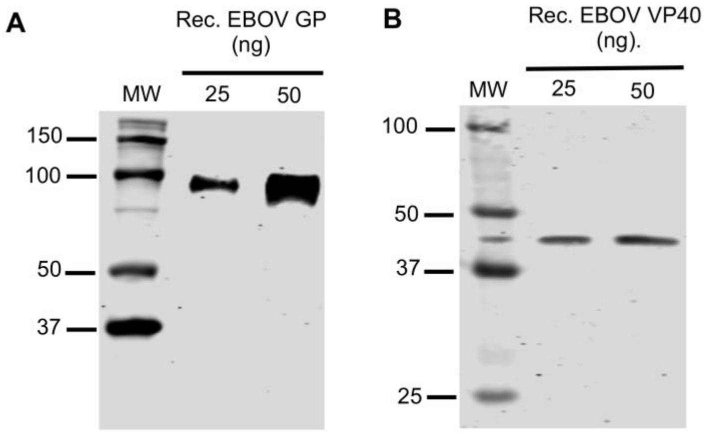

Supplemental Figure S4. Quality of recombinant EBOV GP and VP40 used in this study. Authentication of recombinant EBOV GP ΔTM (A) and recombinant EBOV VP40 (B) by Western blotting using specific antibodies.

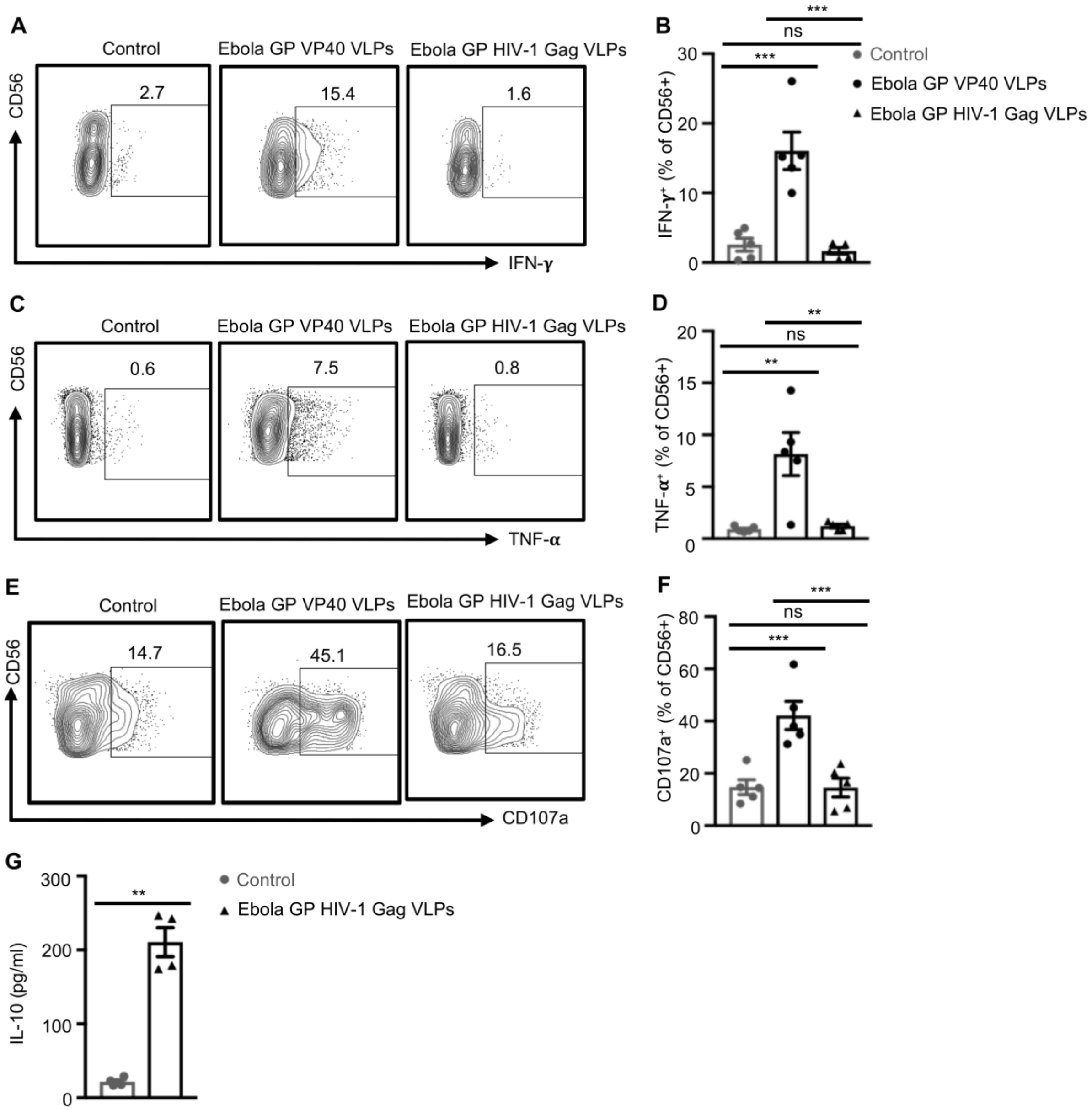

Supplemental Figure S5. **Specificity of EBOV VP40 containing VLPs in inducing activation of NK cells.** PBMCs were left unstimulated or were stimulated with Ebola GP VP40 VLPs (having EBOV VP40 matrix) or Ebola GP HIV-1 Gag VLPs (having HIV-1 Gag matrix) for 24 hours (for cytokines) or 48 hours (for CD107a), stained as in Figures 2 and 3 and data acquired. Representative dot blots showing single and live $CD3^{-}CD56^{+}$ NK cells plotted for IFN-γ, TNF-α and CD107a are shown in (A), (C) and (E), respectively. Summary data from 5 independent experiments performed with PBMCs from 5 donors is shown in (B), (D) and (F), respectively. Results are shown as mean ± SEM. (G) Culture supernatants from the PBMCs from 4 donors, left unstimulated (control) or stimulated with EBOV GP HIV-1 Gag VLPs, were analyzed for IL-10. Results are shown as mean ± SEM. **P < 0.01, ***P < 0.001, ns P > 0.05 as calculated by RM one-way ANOVA followed by Sidak's multiple comparisons test.

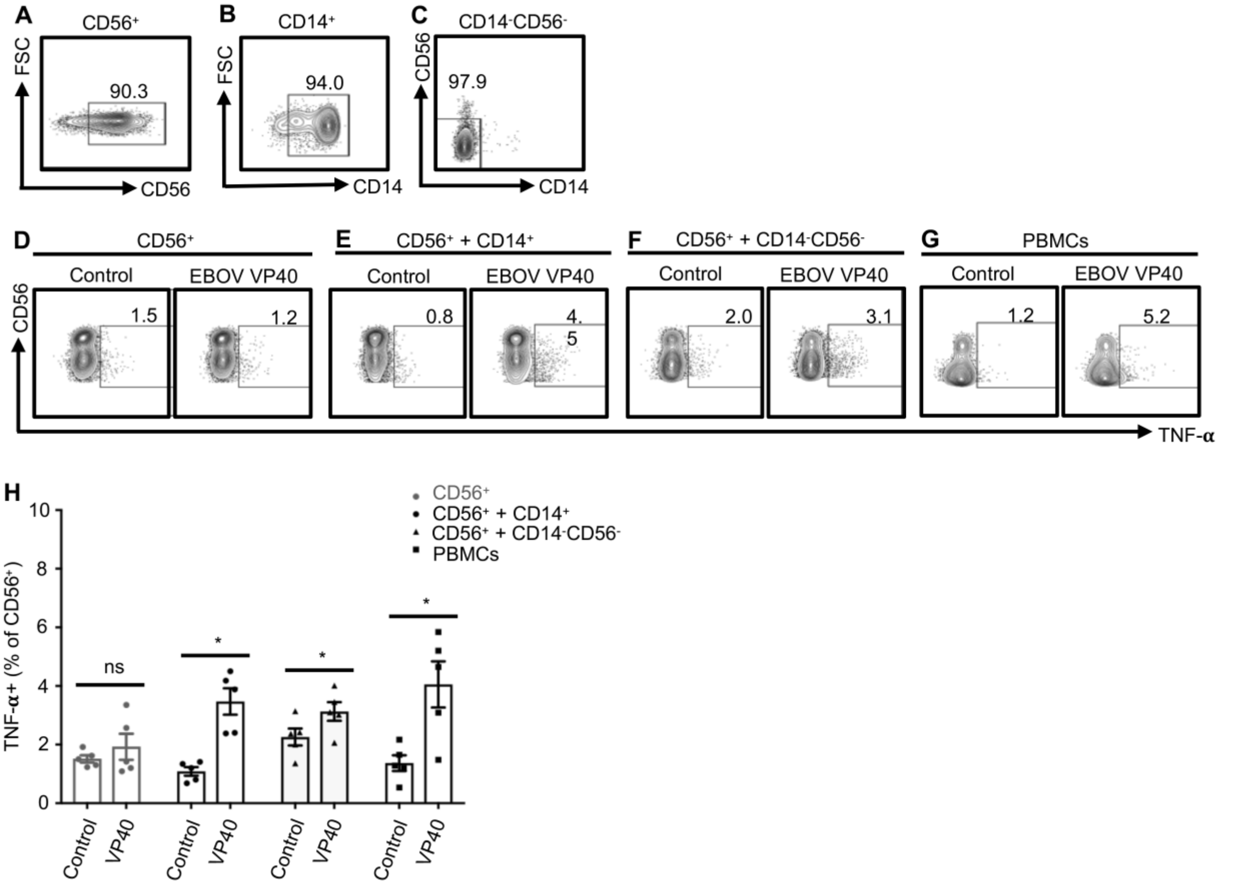

Supplemental Figure S6. CD14$^+$ and CD14$^-$CD16$^-$ accessory cells are required for optimal EBOV VP40-induced TNF-α secretion by the activated NK cells. Purification profiles of isolated CD56$^+$ (A), CD14$^+$ (B) and CD14$^-$CD56$^-$ cells (C). Isolated CD56$^+$ NK cells either alone (D) or in the presence of CD14$^+$ cells (E) or CD14$^-$CD56$^-$ fraction (F), and the parent PBMCs (G) were stimulated with or without EBOV VP40 (5µg/mL) for 24 hours. Cells were analyzed flow cytometrically. Representative dot blots with CD3$^-$CD56$^+$ NK cells plotted for TNF-α are shown. Corresponding summary data from 5 independent experiments performed with cells isolated from 5 donors is shown in (H). Results are shown as mean ± SEM. *$P < 0.05$, ns $P > 0.05$ as calculated by two-tailed paired Student's t-test.

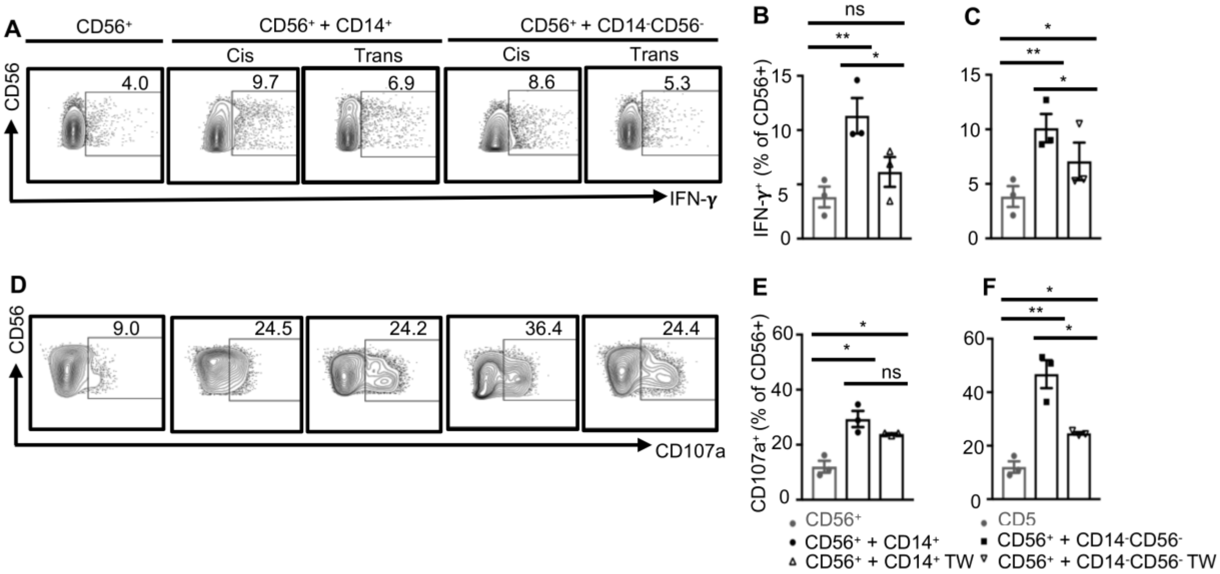

Supplemental Figure S7. Soluble factors as well as cell-cell contact between the CD56⁺ and the accessory cells contribute to the EBOV VP40-induced activation of NK cells. Isolated CD56⁺ NK cells were cultured either alone or in the presence of CD14⁺ or CD14⁻CD56⁻ cells and were stimulated with EBOV VP40 for 24 hours (cytokine secretion) or 48 hours (CD107a). In transwell experiments CD14⁺ cells or CD14⁻CD56⁻ fractions were added on the top of the filter membrane while CD56⁺ NK cells were added to the bottom of the well. EBOV VP40 was added to the accessory cells (top) and GolgiPlug and GolgiStop mix was added to the CD56⁺ NK cells. Cells were stained and analyzed by flow cytometry. Representative dot blots with CD3⁻CD56⁺ NK cells plotted for IFN-γ and CD107a are shown in (A) and (D), respectively. Corresponding summary data from 3 independent experiments performed with cells isolated from 3 donors is shown in (B), (C), (E) and (F). *P < 0.05, **P < 0.01, ns P > 0.05 as calculated by RM one-way ANOVA followed by Holm-Sidak's multiple comparisons test.

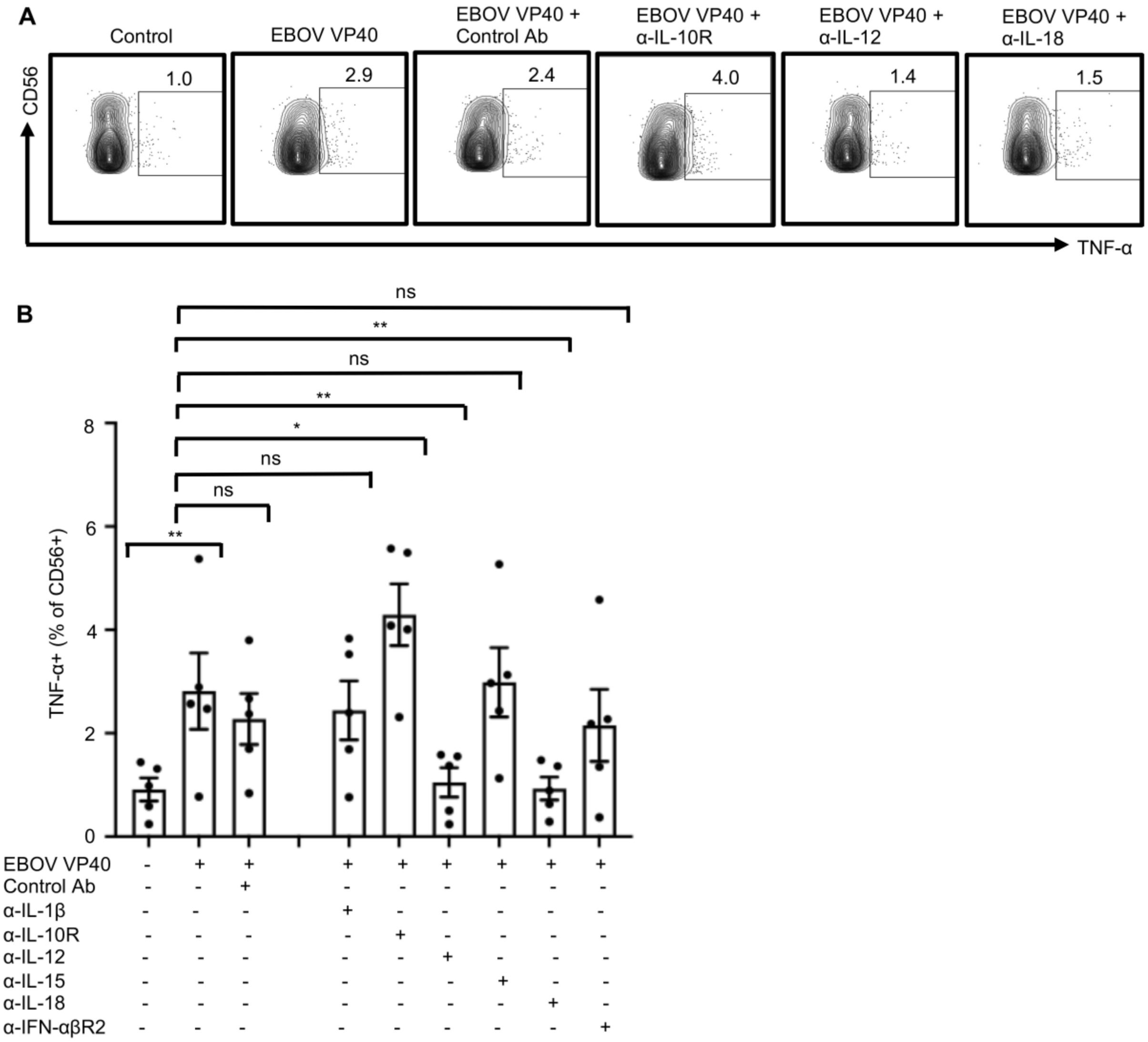

Supplemental Figure S8. IL-12 and IL-18 regulate EBOV VP40-induced NK cell TNF-α responses. PBMCs were cultured, stimulated and analyzed by flowcytometry as in Figure 8. Representative dot blots with $CD3^{-}CD56^{+}$ NK cells plotted for TNF-α are shown in (A). Only dot blots where blocking antibodies showed significant effect compared to the EBOV VP40 are shown. Corresponding summary data from 5 independent experiments performed with cells isolated from 5 donors is shown in (B). Results are shown as mean ± SEM. $^{*}P < 0.05$, $^{**}P < 0.01$, ns $P > 0.05$ as calculated by RM one-way ANOVA followed by Sidak's multiple comparisons test.

Table S1: EBOV GP and EBOV VP40 contents in VLP lots.

Lot #	EBOV GP (μg/mL)	EBOV VP40 (μg/mL)	Ratio (EBOV GP/EBOV VP40)
1	90.01	50.04	1.80
2	106.92	39.77	2.69
3	114.36	76.02	1.50
4	105.66	74.43	1.42
5	121.59	58.85	2.07
All Lots	107.71 ± 11.78	59.82 ± 15.61	1.9 ± 0.51
